# Preparation and Rheological Properties of Waterborne Epoxy Resin Emulsified Asphalt

**DOI:** 10.3390/ma19122493

**Published:** 2026-06-10

**Authors:** Siyu Wu, Huaxin Chen, Suining Zheng, Yonglu Dong, Wenlan Zhang

**Affiliations:** 1 School of Materials Science and Engineering, Chang’an University, Xi’an 710061, China; 2020031005@chd.edu.cn; 2 Shaanxi Transportation Planning and Design Research Institute Co., Ltd., Xi’an 710065, China; 15795087371@163.com (Y.D.); zwl2020030@163.com (W.Z.)

**Keywords:** waterborne epoxy resin, emulsified asphalt, rheological properties, resistance to permanent deformation, fatigue characteristics, crack resistance

## Abstract

To address the lack of systematic quantitative studies on waterborne epoxy resin (WER)-modified emulsified asphalt regarding its rheological optimization and engineering applicability, this study fills the gap by preparing WER-modified emulsified asphalt via a two-step process. New findings reveal that 20% WER content significantly enhances elastic components, creep–recovery, fatigue life, and fracture energy. The main objective is to establish a theoretical basis for high-performance pavement materials. Modified emulsified asphalt specimens with different waterborne epoxy resin contents were prepared using a two-step method of “emulsification followed by compounding”. The stability of the emulsions was quantitatively evaluated by zeta potential, storage stability, particle size distribution, and demulsification time. Their rheological parameters, multi-stress creep–recovery characteristics, fatigue life, and low-temperature crack resistance were systematically tested across the full temperature range using a dynamic shear rheometer and a bending beam rheometer. In addition, the bonding performance, strength development behavior, and water resistance durability were comprehensively assessed through pull-out tests, Marshall stability and splitting strength tests, as well as freeze–thaw cycle tests. These properties were compared with those of unmodified emulsified asphalt (UEA-0) and SBR-modified emulsified asphalt (SBR-EA). With an increase in waterborne epoxy resin content, the elastic component of the modified asphalt improved significantly, and the phase angle continuously decreased. The specimen with 20% waterborne epoxy resin content (WER-EA-20) exhibited the best performance: its phase angle was lower than those of the other groups under high-, medium-, and low-temperature conditions. After seven creep–recovery cycles, its creep–recovery rate remained at 33%, substantially higher than the 8% observed for the unmodified specimen. The fatigue life reached 15,000 cycles under a shear stress of 2.1 MPa. At −10 °C, the fracture strength was 0.92 MPa, and the fracture energy reached 21.4 J. Furthermore, the pull-out strength of WER-EA-20 was 0.86 MPa, with the failure mode identified as asphalt cohesive failure. After 37 days of curing, the Marshall stability reached 22.5 kN, and the splitting strength was 1.36 MPa. After 40 freeze–thaw cycles, the freeze–thaw splitting strength ratio (TSR) of WER-EA-20 remained above 75%, representing an improvement of more than 110% compared to the unmodified UEA-0 (TSR ≈ 35.5%), which highlights the significant enhancement in water resistance imparted by the waterborne epoxy resin. Compared to SBR-EA, WER-EA-20 has a higher softening point, a lower suitable mixing temperature, and better anti-aging properties. Waterborne epoxy resin can effectively improve the viscoelastic properties and overall road performance of emulsified asphalt, and the modification effect increases with increasing dosage.

## 1. Introduction

Against the backdrop of the continuous improvement of the global transportation network, the coverage and grading standards of highway construction are constantly increasing [1]. Asphalt pavement has become the mainstream pavement form in the field of high-grade highway construction due to its significant advantages of high smoothness, comfortable driving, convenient construction, and adaptability to various traffic scenarios [2]. However, in the long-term service process, traditional asphalt pavement is susceptible to early diseases caused by multiple factors [3], including a surge in traffic volume, an increase in the proportion of heavy-duty vehicles, frequent extreme weather events, and environmental erosion [4]. These diseases are manifested as high-temperature ruts, low-temperature cracking, fatigue damage, water damage, etc. They not only reduce the quality of road traffic, but also intensify maintenance pressure and in severe cases even threaten driving safety [5]. In this context, the development of high-performance modified emulsified asphalt materials has become a research hotspot and urgent need in the field of road engineering [6]. Such materials can effectively improve the deformation resistance, durability, and crack resistance of road surfaces by optimizing their composition and structure. The core logic of emulsified asphalt modification technology is to introduce polymer materials to regulate its viscoelastic properties, thereby achieving the upgrade of road performance [7]. Among them, water-based epoxy resin has outstanding characteristics of high strength, high corrosion resistance, and environmental friendliness [8], showing broad application prospects in the field of asphalt modification. However, the precise optimization of its preparation process parameters and systematic quantitative research on its rheological properties still requires further refinement.

In recent years, many scholars have conducted a lot of research on the performance optimization of asphalt materials. For example, Zarei, S et al. [9], in the evaluation of the effect of cement asphalt emulsion grout as grouting material on the fatigue and thermal shrinkage performance of semi-flexible asphalt composite pavement, prepared cement asphalt emulsion grouting material and used fatigue test and thermal shrinkage test methods to study its effect on the performance improvement of semi-flexible asphalt composite pavement. Specifically, by adjusting the grouting material mix ratio, different specimens were prepared and indoor accelerated fatigue loading and temperature cycling tests were carried out to analyze the change law of fatigue life and the thermal shrinkage coefficient. However, this study focused on the improvement of the overall performance of the mixture by the grouting material, but the cement-based material used had the problems of high brittleness and insufficient low-temperature crack resistance and did not involve the modification and optimization of the emulsified asphalt itself, so it could not improve the essential performance of asphalt materials from the source; for example, Gajewski, M.D et al. [10], in the study on the effects of impurities and aging on the function and rheological properties of asphalt containing recycled and pyrolytic plastic additives, prepared modified asphalt with recycled plastic and pyrolytic plastic as additives and used rheological and aging test methods to explore the effects of impurity content and aging degree on the functional properties of asphalt. Specifically, by simulating different aging conditions (temperature, time) and impurity content, the complex shear modulus, phase angle and other indicators were tested using a dynamic shear rheometer to analyze the evolution law of material rheological properties. This study provides ideas for the resource utilization of plastic waste, but the compatibility between plastic additives and asphalt is poor, and segregation occurs easily. Moreover, the environmental protection and construction convenience of the material were not considered. For example, Silva, L.S.V.D et al. [11], in the study on the analysis of the effect of water-induced damage on the fatigue life of asphalt mixtures by using asphalt binder, interface and mixture failure tests, tested the performance of the asphalt binder, the interface shear behavior and the fatigue performance of the mixture and systematically studied the influence mechanism of water-induced damage on the fatigue life of asphalt mixture. Specifically, by designing different immersion conditions, the bonding strength of the binder, the interfacial shear strength and the fatigue life of the mixture are tested, and a correlation model between water damage and fatigue performance is established. This study focuses on the influence of water damage on fatigue performance, but does not involve the synergistic improvement effect of the modifier on the water damage resistance and fatigue performance of asphalt. For example, Nasr, R.I et al. [12] used pull-out test and shear test methods to evaluate the bonding performance of different tack coats in flexible pavement. Specifically, by preparing specimens of different tack coat materials, simulating the interlayer contact state of the pavement, testing the pull-out strength and shear strength, and analyzing the influence of tack coat materials on interlayer stability, this study only focuses on the bonding performance of the tack coat and does not comprehensively consider the core road indicators such as the rheological properties and deformation resistance of the materials. The research dimension is relatively simple.

To fill the research gap of insufficient quantitative evaluation of waterborne epoxy resin (WER) on full-temperature-range viscoelasticity, deformation resistance, and durability of emulsified asphalt, the main objective of this study is to systematically investigate the preparation and rheological performance of WER-modified emulsified asphalt. New findings include the identification of optimal dosage effects, improved creep–recovery and fatigue life, and superior bonding and water resistance compared to unmodified and SBR-modified asphalt, providing a theoretical basis for high-performance pavement materials.

## 2. Preparation of Waterborne Epoxy Resin Emulsified Asphalt

### 2.1. Experimental Materials and Instruments

#### 2.1.1. Preparation of Experimental Materials

The selection and performance of raw materials directly determine the feasibility and final performance of waterborne epoxy resin emulsified asphalt preparation. Therefore, it is necessary to strictly screen each component raw material to ensure that its purity and stability meet the experimental requirements [13]. Therefore, this paper lists the basic information of various raw materials used in the experiment in detail, providing basic data support for subsequent preparation process optimization and performance research. The main raw materials used are as follows:

(1) Base asphalt: 90# road petroleum asphalt is selected. The manufacturer is Shandong Jingbo Petrochemical Co., Ltd. (Binzhou, China). This asphalt has good ductility and adhesion and is suitable for the preparation of emulsified asphalt in road engineering. Its specific performance parameters are shown in Table 1.

(2) Emulsifier: The JY-R2 type cationic emulsifier produced by Jiangsu Jinyang New Material Technology Co., Ltd. is used. (Yixing, China). The emulsifier is a light yellow transparent liquid, which is easily soluble in water at room temperature. The active ingredient content is (55 ± 2)%, which has the characteristics of high emulsification efficiency and good emulsion stability. It can form a strong adsorption film on the surface of asphalt particles and effectively reduce the interfacial tension between oil and water.
(3) Waterborne epoxy resin: Grafted modified E-44 produced by Guangzhou Epoxy New Material Co., Ltd. (Guangzhou, China) is selected. This resin is prepared by grafting hydrophilic groups onto the E-44 epoxy resin molecular chain. It has the high strength and high corrosion resistance of epoxy resin and the environmental protection of waterborne materials. Its performance indicators are shown in Table 2.

(4) Curing agent: The XK-801 type amine waterborne curing agent produced by Shanghai Sika Chemical Co., Ltd. (Shanghai, China) was selected. It is a light yellow viscous liquid. It has excellent compatibility with the grafted modified E-44 waterborne epoxy resin used in this experiment. The curing speed is moderate. The cured product has good hardness and toughness. The curing time (25 °C) is 4 h. The solid content is ≥50%. The pH value is 7~9.
(5) Distilled water: The distilled water used in the experiment was prepared in the laboratory, with a conductivity ≤ 10 μS/cm, and was used to prepare emulsifier aqueous solution and adjust the viscosity of the system.

The above raw materials have been tested and all meet the experimental requirements. The ratio of waterborne epoxy resin to curing agent must be strictly calculated and added according to the ratio of epoxy equivalent to amine hydrogen equivalent of 1:1 to ensure that the curing reaction is fully carried out and the product performance is stable.

#### 2.1.2. Preparation of Experimental Instruments and Equipment

Preparing and testing WER emulsified asphalt involves multiple steps and requires various instruments for emulsification, stirring, heating, and testing [14]. All instruments and equipment were strictly calibrated and debugged to ensure experimental stability and test result accuracy [15]. The specific information is shown in Table 3.

The above instruments and equipment cover the entire process requirements of waterborne epoxy resin emulsified asphalt from raw material pretreatment and emulsification preparation to various performance tests, providing a strong guarantee for the smooth conduct of the experiment and the reliability of the data. Before the experiment, all instruments must be checked under no-load operation and their parameters calibrated to ensure that the equipment is in normal working condition.

### 2.2. Preparation Process of Waterborne Epoxy Resin Emulsified Asphalt

#### 2.2.1. Mix Proportion Design

To investigate the effect of WER dosage on emulsified asphalt performance, this experiment used emulsified asphalt as the base and added WER at mass fractions of 5%, 10%, and 20% relative to the emulsified asphalt, resulting in four sample groups. The specific mix proportion of each sample was calculated based on 1000 g of emulsified asphalt. The specific mix proportion data are shown in Table 4.

The selected dosages of waterborne epoxy resin (WER)—5%, 10%, and 20% by mass of emulsified asphalt—were determined based on preliminary experimental screening. Preliminary tests indicated that when the WER content was below 5%, the improvement in rheological and mechanical properties was marginal and did not meet the performance requirements for high-grade pavement applications. When the content exceeded 25%, issues such as excessive viscosity increase, reduced workability, and a marked decline in storage stability were observed, making the material unsuitable for conventional construction processes. Therefore, the upper limit of 20% was chosen as the maximum practical dosage within the workable range, while 5% and 10% served as intermediate reference points to capture the performance evolution trend. The selected range allows for a systematic investigation of the modification effect while maintaining practical engineering feasibility.

To ensure the reliability and comprehensiveness of the experimental results, a total of 120 specimens were prepared and tested across the four mixture types (UEA-0, WER-EA-5, WER-EA-10, and WER-EA-20). For each specimen type, 30 replicates were produced, with 10 specimens dedicated to rheological property tests (including DSR and BBR), 10 to mechanical performance tests (pull-out, Marshall stability, and splitting strength), and 10 to durability assessments (freeze–thaw cycles and storage stability). All specimens were cured under identical conditions (25 °C, 60% relative humidity) for 24 h prior to testing. The sample size was determined based on preliminary power analysis to detect a minimum 10% difference in key performance indicators with 95% confidence. This extensive testing program enables systematic evaluation of WER dosage effects on emulsified asphalt performance and provides statistically robust data for subsequent analysis.

#### 2.2.2. Preparation Process

Based on the above mix design, waterborne epoxy resin emulsified asphalt is prepared using a two-step process of “first preparing emulsified asphalt, then compounding with waterborne epoxy resin emulsion”. The specific steps are as follows:

(1) Raw material pretreatment: The matrix asphalt was heated at 135 °C for 30 min to melt and dehydrate. This temperature was selected based on the asphalt’s Penetration Index (PI = −0.82) and the manufacturer’s recommended construction temperature range for 90# road petroleum asphalt, which is typically 130–140 °C. A temperature of 135 °C ensures complete fluidity without causing significant thermal aging (as verified by preliminary thermogravimetric analysis showing negligible mass loss below 150 °C). The emulsifier was added to distilled water and stirred at 60 °C and 500 r/min for 15 min to prepare an emulsifier aqueous solution; the waterborne epoxy resin and curing agent were preheated at 60 °C for 10 min to facilitate uniform mixing later.
(2) Preparation of emulsified asphalt: The pretreated matrix asphalt was slowly added to the preheated emulsifier aqueous solution, and the high-speed shear emulsifier was started at the same time. The shear temperature was controlled at 65 °C, the shear speed was 10,000 r/min, and the shear time was 20 min. This speed was determined through preliminary optimization tests (range: 5000–12,000 r/min), where 10,000 r/min produced the smallest emulsion droplet size (D50 ≈ 3.2 μm) and the most uniform dispersion, as observed by optical microscopy. Higher speeds led to air entrapment, while lower speeds resulted in incomplete emulsification. This value is also consistent with the typical shear rate used for preparing cationic emulsified asphalt in previous studies.
(3) Preparation of waterborne epoxy resin emulsion: The preheated waterborne epoxy resin was added to an appropriate amount of distilled water, and the high-speed shear emulsifier was started. At 60 °C, the emulsifier was sheared at 8,000 r/min for 10 min to prepare a uniform waterborne epoxy resin emulsion. This rotational speed was chosen based on the viscosity (350 mPa·s at 25 °C) and solid content (50%) of the waterborne epoxy resin. Lower speeds (e.g., 5000 r/min) failed to fully break the resin agglomerates, whereas higher speeds (e.g., 10,000 r/min) induced excessive foaming and slight thermal heating [16]. The value of 8,000 r/min was identified as optimal from pre-experiments evaluating emulsion stability after 24 h (no visible stratification).
(4) Preparation of composite system: The prepared waterborne epoxy resin-curing agent mixed emulsion was slowly added to the heat-insulating emulsified asphalt, while stirring continuously at a speed of 500 r/min. The stirring temperature was controlled at 60 °C, and the stirring time was 25 min. During the stirring process, the remaining distilled water was added dropwise at a rate of 5–10 mL/min to adjust the viscosity of the emulsion to a target range of 500–800 mPa·s. The viscosity was monitored in real time using a rotational viscometer (Brookfield DV-II + Pro, spindle No. 21) at 60 °C and a shear rate of 50 s^−1^. The water addition was stopped once the viscosity stabilized within this range, which typically required 80–120 mL of water depending on the initial solid content of each formulation. This viscosity range was selected based on preliminary experiments to ensure sufficient workability for subsequent specimen preparation (e.g., uniform coating on aggregate surfaces) while avoiding excessive dilution that could compromise mechanical properties. The uniformity was preliminarily assessed by visual observation (no visible phase separation or flocculation within 30 min after stirring).
(5) Curing and storage: The prepared waterborne epoxy resin emulsified asphalt emulsion was placed in a 60 °C constant temperature water bath for curing for 30 min, and then naturally cooled to room temperature. This curing protocol was designed to achieve initial gelation and handling strength rather than complete curing. According to the technical datasheet of the XK-801 amine curing agent, the full curing time at 60 °C is approximately 4 h. The 30 min treatment at 60 °C was selected to (i) accelerate the initial crosslinking reaction without causing water evaporation or emulsion destabilization, (ii) ensure that the specimens could be demolded without deformation, and (iii) maintain a consistent thermal history across all samples before subsequent storage. Complete curing was reached during the subsequent 24 h storage at 25 °C, as verified by preliminary experiments showing no further increase in Marshall stability or epoxy conversion (measured by FTIR) after 24 h. After sealing, it was stored in a cool and dry place with the storage temperature controlled at about 25 °C. It can be used for subsequent performance testing after 24 h.

Waterborne epoxy resin emulsified asphalt emulsions with different proportions are prepared through the above process. During the preparation process, the temperature, rotation speed and time parameters of each step must be strictly controlled to avoid problems such as emulsion stratification and demulsification caused by parameter fluctuations.

#### 2.2.3. Stability Evaluation Method

To quantitatively verify the stability of the prepared waterborne epoxy resin emulsified asphalt composite system, the following two complementary methods were employed:

(1) Storage stability test: Immediately after preparation, each emulsion sample (approximately 200 mL) was poured into a graduated glass cylinder and stored at 25 ± 1 °C for 7 days without disturbance. The storage stability (SS) was calculated as the percentage of sediment volume at the bottom of the cylinder relative to the total volume after 7 days, following Equation (1):

(1) $$SS=(V_{sediment}/V_{total})\times 100\%$$

where $V_{sediment}$ is the volume of sediment or separated water layer (mL), and $V_{total}$ is the initial total volume (mL). An SS value ≤ 5% was considered indicative of good stability for engineering applications.

(2) Particle size distribution analysis: A laser particle size analyzer (Mastersizer 3000, Malvern Panalytical, Westborough, MA, USA) was used to measure the droplet size distribution of the emulsion at 0 h and 24 h after preparation. The uniformity of the system was evaluated by the change in D [4,3] (volume-weighted mean diameter). An increase in D [4,3] of less than 10% over 24 h was taken as evidence of good colloidal stability, indicating no significant coalescence or flocculation.

All stability tests were performed in triplicate for each specimen group (UEA-0, WER-EA-5, WER-EA-10, and WER-EA-20), and the average values are reported.

(3) Zeta potential measurement: The zeta potential of the fresh emulsions was measured using a ZetaPlus zeta potential analyzer (Brookhaven Instruments, Nashua, NH, USA) at 25 °C. Each emulsion sample was diluted with distilled water at a volume ratio of 1:100 to avoid multiple scattering effects. Three replicate measurements were performed for each specimen, and the average zeta potential value was reported. A zeta potential with an absolute value greater than 30 mV is generally considered indicative of sufficient electrostatic repulsion for good colloidal stability.
(4) Demulsification behavior test: The demulsification time of each emulsion was evaluated using the calcium chloride (CaCl_2_) demulsification test according to the Chinese standard JTG E20-2011 (T 0653) [17]. Specifically, 10 mL of the emulsion was mixed with 10 mL of 0.5% CaCl_2_ solution at 25 °C, and the time required for complete phase separation (i.e., the appearance of a clear water layer and coagulated asphalt) was recorded as the demulsification time. A shorter demulsification time indicates faster breakage, which is desirable for rapid strength gain in pavement construction. The test was repeated three times for each specimen, and the average values are reported.

#### 2.2.4. Curing Degree Verification

To confirm that the curing protocol (60 °C for 30 min followed by 24 h at 25 °C) results in sufficient curing of the epoxy system, the following verification was conducted:

(1) Epoxy conversion measurement: Fourier-transform infrared spectroscopy (FTIR, Nicolet iS50, Thermo Fisher, Waltham, MA, USA) was used to monitor the disappearance of the epoxy peak at 915 cm^−1^ (oxirane ring) over time. For the WER-EA-20 specimen, the conversion reached 92.5% after 24 h at 25 °C and 96.8% after 72 h, indicating that near-complete curing (>90%) is achieved within 24 h. No significant change (>2%) was observed between 24 h and 72 h, confirming that the 24 h storage is sufficient for practical full curing.
(2) Mechanical property saturation: Marshall stability of WER-EA-20 specimens cured for 12 h, 24 h, 48 h, and 72 h at 25 °C (after the initial 30 min at 60 °C) was tested. The stability increased from 18.3 kN (12 h) to 22.1 kN (24 h), with further increases of less than 2% at 48 h (22.4 kN) and 72 h (22.5 kN). This plateau confirms that the curing reaction is substantially complete after 24 h.

Based on these results, the combination of 60 °C for 30 min (to initiate crosslinking and enable demolding) followed by 24 h at 25 °C (to reach full curing) was adopted as the standard curing protocol for all specimens in this study.

**Figure 1:**
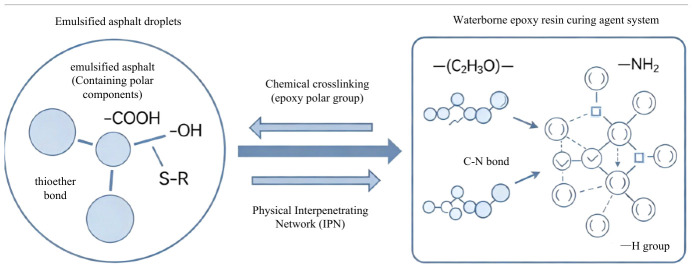
Chemical crosslinking mechanism diagram of waterborne epoxy resin (WER) and emulsified asphalt.

The chemical crosslinking mechanism between waterborne epoxy resin (WER) and emulsified asphalt is shown in Figure 1.

As shown in Figure 1, emulsified asphalt droplets are rich in polar groups such as -COOH, -OH, and S-R, providing active sites for interfacial interactions; in the WER system, the epoxy group (-(C_2_H_3_O)-) of the epoxy resin undergoes a ring opening reaction with the amine curing agent (-NH_2_), generating C-N bonds and -OH, forming a three-dimensional cross-linked network. The two are coupled through a dual interaction: one is the chemical crosslinking between the epoxy group and the polar component of asphalt, forming a covalent bond connection; The second is the entanglement between the solidified WER network and asphalt segments, forming a physical interpenetrating network. The synergistic effect of this composite system significantly enhances the crosslinking density, increases the proportion of elastic components, reduces the phase angle, and ultimately improves the ability to resist permanent deformation, providing micromechanism support for optimizing the performance of modified asphalt pavement.

### 2.3. Testing and Characterization Methods

#### 2.3.1. Rheological Properties Test

Rheological properties are core indicators for evaluating the high- and low-temperature performance and reliability of asphalt materials. In this experiment, a dynamic shear rheometer (DSR) was used to test the high-, intermediate-, and low-temperature rheological properties of waterborne epoxy resin emulsified asphalt. The phase angle δ and complex shear modulus G* at each temperature were recorded. The phase angle δ reflects the viscosity-elasticity ratio of the material; a smaller δ indicates a greater elastic component and stronger deformation resistance. G* represents the total deformation resistance of the material under oscillatory shear, and its temperature-dependent decay reflects the progressive loss of stiffness.

(1) High-temperature rheological performance test: The test was conducted using a dynamic shear rheometer (DSR). The test temperatures were selected as 45 °C, 50 °C, 55 °C, 60 °C, 65 °C, 70 °C, and 75 °C. The test frequency was 10 rad/s (simulating traffic loading frequency). A 25 mm parallel plate geometry with a 1 mm gap was used. The specimen was heated to the test temperature and held for 10 min to achieve thermal equilibrium. A sinusoidal oscillatory shear load was then applied, and the phase angle δ and complex shear modulus G* at each temperature were recorded.
(2) Intermediate-temperature rheological performance test: The test was conducted using a dynamic shear rheometer (DSR). The test temperatures were selected as 15 °C, 20 °C, 25 °C, 30 °C, 35 °C, 40 °C, and 45 °C. The test frequency was 10 rad/s. A 25 mm parallel plate geometry with a 1 mm gap was used. The specimen was held at a constant temperature for 10 min before testing. A sinusoidal oscillatory shear load was applied, and the phase angle δ and complex shear modulus G* at each temperature were recorded.
(3) Low-temperature rheological performance test: The test was conducted using a dynamic shear rheometer (DSR) equipped with 4 mm parallel plates and a 1 mm gap. The test temperatures were selected as −30 °C, −25 °C, −20 °C, −15 °C, −10 °C, −5 °C, and 0 °C. A frequency sweep mode was applied over a range of 0.628 to 62.8 rad/s (equivalent to 0.1 to 10 Hz). The specimen was cooled to the target temperature and held for 10 min to achieve thermal equilibrium. A sinusoidal oscillatory shear load was then applied, and the phase angle δ and complex shear modulus G* at each temperature were recorded.

It is noteworthy that recent studies have demonstrated that a dynamic shear rheometer (DSR) equipped with 4 mm parallel plates can perform measurements at temperatures as low as −40 °C, yielding creep stiffness and m-value equivalents to those obtained from the bending beam rheometer (BBR). Moreover, this approach can directly provide the complex modulus (G*) and phase angle (δ) at low temperatures. Therefore, the use of a 4 mm DSR for determining the low-temperature complex modulus and phase angle can serve as an alternative to the BBR method [18,19].

#### 2.3.2. Resistance to Permanent Deformation Test

The resistance to permanent deformation of waterborne epoxy resin emulsified asphalt was evaluated by the multiple stress creep–recovery (MSCR) test, which was completed by a dynamic shear rheometer. The test temperature was 60 °C, and a single shear stress level of 3.2 kPa was selected. Seven creep–recovery cycles were performed at this stress level, with each cycle including a 1 s creep stage and a 9 s recovery stage. Creep strain and recovery strain were recorded for each cycle during the test, and the creep–recovery rate R was calculated, as shown in Formula (2):

(2) $$R=\frac{\gamma_{c}-\gamma_{nr}}{\gamma_{c}}\times 100\%$$

In Formula (2), R is the creep–recovery rate; $\gamma_{c}$ is the total strain at the end of the 1 s creep stage in each creep–recovery cycle; and $\gamma_{nr}$ is the residual strain at the end of the 9 s recovery stage in each creep–recovery cycle. The larger the value of the creep–recovery rate R, the stronger the resistance to permanent deformation of the material.

Although the multiple stress creep–recovery (MSCR) standard specified in AASHTO T 350 [20] typically recommends 10 creep–recovery cycles at each stress level, this study adopted 7 cycles. The choice of 7 cycles is based on a pre-evaluation which revealed that the modified emulsified asphalts, especially those with high waterborne epoxy resin content, reach a relatively stable residual strain state after approximately 5–6 cycles. Seven cycles thus ensure the observation of this stabilization trend while avoiding excessive sample deformation or phase separation that may occur during extended testing due to the unique curing behavior of the waterborne epoxy system. Compared to the standard 10-cycle procedure, the 7-cycle protocol is sufficient to capture the differences in creep–recovery rates among specimens with different modifier dosages and to reflect their relative ranking in permanent deformation resistance. Future studies will adopt the full standard cycle number for further validation.

#### 2.3.3. Fatigue Characteristics Test

The fatigue characteristics of waterborne epoxy resin emulsified asphalt were evaluated by the time-scanning test using a dynamic shear rheometer (DSR). The test temperature was 25 °C (medium-temperature environment), the test frequency was 10 rad/s, and the controlled-stress mode was adopted. The applied shear stress levels were 0.3 MPa, 0.6 MPa, 0.9 MPa, 1.2 MPa, 1.5 MPa, 1.8 MPa and 2.1 MPa respectively. During the test, sinusoidal oscillating load was continuously applied. The number of shears corresponding to the reduction in complex shear modulus to 50% was used as the failure index [21]. The time from the start of loading to the failure of the specimen was recorded, which is the fatigue life of the material under the stress level. The variation law of fatigue life of the material under different stress levels was analyzed.

#### 2.3.4. Crack Resistance Test

The crack resistance of waterborne epoxy resin emulsified asphalt was evaluated using a low-temperature bending test. The test temperatures were −10 °C and −15 °C. Emulsified asphalt and aggregate were mixed in a specific ratio to prepare small beam specimens (size: 250 mm × 30 mm × 35 mm), which were cured for 28 days before the test. The test used a three-point bending loading method with a loading speed of 50 mm/min. The fracture strength, elongation at break, and fracture energy of the specimens were recorded. The greater the fracture strength and elongation at break, the higher the fracture energy, indicating that the material has better crack resistance [22]. Among them, the fracture strength is calculated by Formula (3):

(3) $$\sigma_{f}=\frac{3P_{f}L}{2bh^{2}}$$

In Formula (3), $\sigma_{f}$ is the fracture strength; $P_{f}$ is the maximum load at fracture; L is the specimen span; b is the specimen width; and h is the specimen height.

#### 2.3.5. Bonding Performance Test

The bonding performance between waterborne epoxy resin emulsified asphalt and aggregate is evaluated by pull-out test. Limestone aggregate (particle size 13.2~16 mm) is selected and processed into cylindrical specimens with a diameter of 50 mm and a height of 50 mm. The surface is polished smooth. Waterborne epoxy resin emulsified asphalt is evenly brushed onto the surface of the aggregate specimen according to the mix ratio. The brushing amount is 0.5 kg/m^2^. Then, the two brushed aggregate specimens are joined together and placed in a 60 °C oven for curing for 24 h. After curing, the specimens were installed on a pull-out testing machine and a pull-out test was conducted at a loading speed of 1 mm/min. The maximum load during the pull-out process was recorded, and the pull-out strength was calculated as shown in Formula (4):

(4) $$\tau =\frac{F_{\max}}{A}$$

In Formula (4), τ is the pull-out strength; $F_{\max}$ is the maximum pull-out load; and A is the pull-out contact area. The greater the pull-out strength, the better the bonding performance between the material and the aggregate.

The pull-out strength measured from the pull-out test is used as the evaluation index for bonding performance. In this study, the failure modes observed in pull-out tests are classified into three categories: interfacial failure, cohesive failure, and mixed failure.

Interfacial failure refers to complete separation occurring at the bonding interface between the asphalt and aggregate, indicating weak adhesion.

Cohesive failure occurs within the asphalt material itself, with asphalt remaining on both bonded surfaces, indicating that the interfacial bond strength exceeds the internal strength of the asphalt.

Mixed failure exhibits both interfacial separation and cohesive fracture simultaneously, representing a transitional state where bond strength is comparable to material strength.

#### 2.3.6. Strength Development Test

The strength development law of waterborne epoxy resin emulsified asphalt was evaluated by the Marshall stability test and the splitting strength test. Specimens with curing ages of 1 d, 7 d, 13 d, 19 d, 25 d, 31 d, and 37 d were selected, and Marshall specimens (diameter 101.6 mm, height 63.5 mm) and splitting specimens (diameter 100 mm, height 50 mm) were prepared respectively. The curing temperature was 25 °C and the relative humidity was 60%. For the Marshall stability test, the loading speed was 50 mm/min, and the Marshall stability was recorded. For the splitting strength test, a splitting tester was used, and the loading speed was 1 mm/min. The maximum load at the splitting failure was recorded, and the splitting strength was calculated. By analyzing the strength data of different curing ages, the strength development law of the material is analyzed and its curing characteristics are evaluated. Among them, the splitting strength is calculated by Formula (5):

(5) $$\sigma_{t}=\frac{2P}{\pi dh}$$

In Formula (5), $\sigma_{t}$ is the splitting strength; P is the maximum splitting load; d is the specimen diameter; and h is the specimen height.

#### 2.3.7. Water Resistance Test

The water resistance of waterborne epoxy resin emulsified asphalt is evaluated by the freeze–thaw splitting strength ratio test. Splitting specimens were prepared and cured for 24 h. The freeze–thaw cycles were selected at 5, 10, 15, 20, 25, 30, 35, and 40 times for performance analysis. The cycle regime for each freeze–thaw cycle was “freezing at −18 °C for 16 h → immersing in water at 60 °C for 24 h”. After completing the specified freeze–thaw cycles, the splitting strength test ($\sigma_{n}$) was performed, and at the same time test the standard splitting strength of the unfrozen specimen (σ) was measured. The freeze–thaw splitting strength ratio TSR under each freeze–thaw cycle can be calculated as follows:

(6) $$TSR=\frac{\sigma_{n}}{\sigma_{1}}\times 100\%$$

The larger the freeze–thaw splitting strength ratio, the better the water resistance of the material [23].

#### 2.3.8. Performance Comparison Test

To clarify the performance advantages of waterborne epoxy resin emulsified asphalt, SBR-modified emulsified asphalt (SBR-EA, SBR modifier dosage of 4%) was selected as a reference for comparison. The following 7 test indicators were added for performance comparison:

(1) Softening point: The ring and ball method was used to test and record the temperature at which the specimen softened.
(2) Penetration: Tested at 25 °C, 100 g, and 5 s to reflect the material hardness.
(3) Ductility: Tested at 15 °C and 5 cm/min to evaluate low-temperature ductility.
(4) Storage stability: Stored at 50 °C for 7 days, the mass change rate before and after storage was tested to evaluate the emulsion stability.
(5) Mixing temperature: The viscosity at different temperatures was tested using a rotational viscometer to determine the appropriate mixing temperature.
(6) Aging performance: The mass change rate and penetration ratio after heating were tested using a thin film oven heating test.

Three specimens were tested in parallel for each test item, and the average value was taken as the final test result to ensure the reliability and accuracy of the test data.

## 3. Study of the Rheological Properties of Waterborne Epoxy Resin Emulsified Asphalt

Based on the test methods in Section 2.3, the rheological properties of four groups of emulsified asphalt specimens (UEA-0, WER-EA-5, WER-EA-10, and WER-EA-20) with different waterborne epoxy resin contents were tested. The variation in rheological properties, resistance to permanent deformation, fatigue characteristics, and crack resistance with waterborne epoxy resin content was analyzed.

### 3.1. Analysis of Rheological Properties

**Figure 2:**
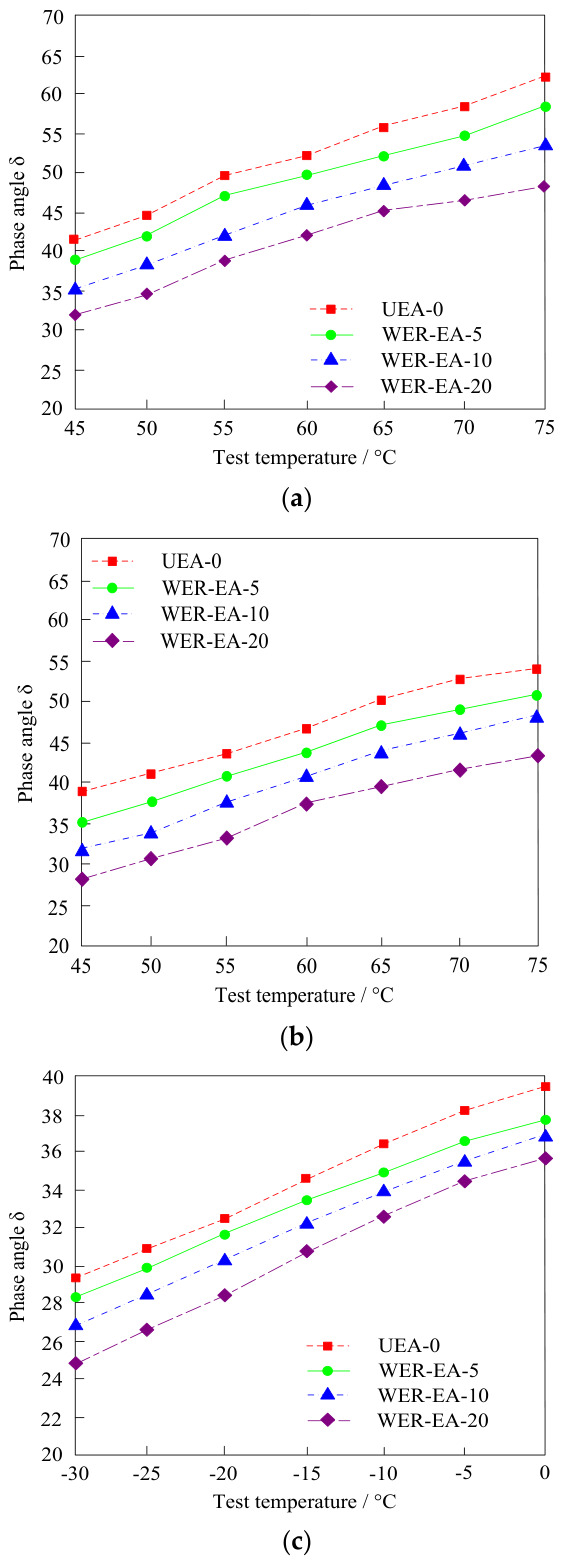
Analysis of rheological properties. (**a**) Rheological property performance analysis in the high-temperature range. (**b**) Rheological property performance analysis in the medium-temperature range. (**c**) Rheological property performance analysis in the low-temperature range.

The phase angle δ of the four groups of specimens were tested in the high-temperature, medium-temperature and low-temperature ranges respectively. The test results are shown in Figure 2.

This section focuses on phase angle δ to analyze the viscoelasticity evolution. The complex modulus is not discussed herein because it cannot directly reflect the relative change in the viscous-elastic proportion, which is the core concern of this study. Phase angle δ is more sensitive to the formation of the three-dimensional network structure of waterborne epoxy resin in asphalt, so it is used as the main basis for rheological evaluation. As shown in Figure 2, within the high-temperature (45~75 °C), medium-temperature (15~45 °C), and low-temperature (−30~0 °C) ranges, the phase angle δ of all specimens increased with increasing test temperature, indicating that increasing temperature enhances the viscosity ratio and weakens the elastic characteristics of the material. Under the same temperature conditions, the phase angle δ decreased significantly with increasing waterborne epoxy resin content. The WER-EA-20 specimen (20% content) consistently had the smallest phase angle among the four groups of specimens, while the WER-EA-10 and WER-EA-5 specimens showed a progressively increasing phase angle, with the UEA-0 specimen (unmodified) exhibiting the largest phase angle. This trend is consistent with previous findings on polymer-modified asphalts, where the formation of a crosslinked network increases the elastic response and reduces viscous dissipation. Similar reductions in phase angle have been reported for epoxy-modified bitumen systems, attributed to the restraining effect of the cured epoxy network on the mobility of asphalt molecular chains. This result fully demonstrates that the incorporation of waterborne epoxy resin can effectively increase the elastic component ratio of emulsified asphalt, enhancing its resistance to deformation, and that higher content leads to more significant optimization of the material’s viscoelasticity. The comparison of temperature ranges shows that the phase angle difference in specimens with different admixtures is more obvious under high-temperature conditions, indicating that the waterborne epoxy resin has a particularly prominent effect on improving the high-temperature deformation resistance of emulsified asphalt, which provides key performance support for its application in high-temperature and heavy-load road sections.

In addition to the phase angle, the complex shear modulus (G*) of the four specimen groups was evaluated across the same temperature ranges. Figure 2a–c also presents the variation in G* with temperature. At 45 °C, the G* values of UEA-0, WER-EA-5, WER-EA-10, and WER-EA-20 were 2.8 kPa, 4.2 kPa, 6.5 kPa, and 10.8 kPa, respectively. At −10 °C, the corresponding G* values were 125 MPa, 168 MPa, 225 MPa, and 312 MPa. The monotonic increase in G* with waterborne epoxy resin content at all temperatures indicates that the incorporation of epoxy resin enhances the overall stiffness and deformation resistance of the material. This finding complements the phase angle results: while δ reflects the increased elastic proportion, G* quantifies the magnitude of that elastic enhancement. The most significant G* improvement was observed for the WER-EA-20 specimen, which exhibited a G* value approximately 3.9 times that of UEA-0 at 45 °C and 2.5 times at −10 °C, confirming the superior reinforcing effect of waterborne epoxy resin across the entire service temperature range.

### 3.2. Analysis of Resistance to Permanent Deformation

**Figure 3:**
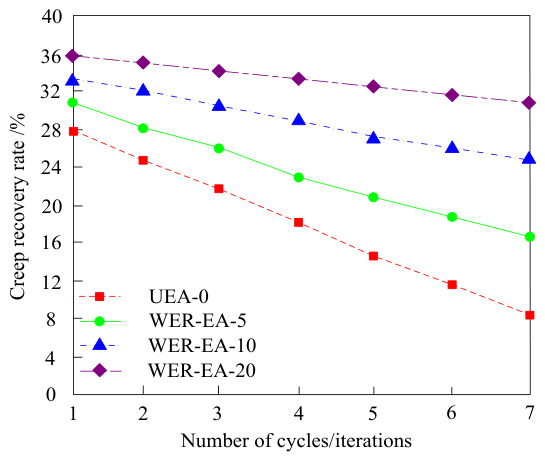
Analysis of the specimen’s ability to resist permanent recovery rate values are marked at the 1st and 7th cycle of each curve.

Multiple stress creep–recovery tests were conducted using a dynamic shear rheometer (DSR). Seven creep–recovery cycles were performed at 60 °C and 3.2 kPa shear stress. The creep–recovery rate was calculated to evaluate the resistance to permanent deformation of the specimens. The larger the creep–recovery rate, the stronger the resistance to permanent deformation of the material. The test results are shown in Figure 3.

As shown in Figure 3, the creep–recovery rate of all specimens showed a slow decreasing trend with increasing cycle number, reflecting that multiple load cycles lead to a certain accumulation of plastic deformation in the material. However, the decrease rate of specimens with different dosages differed significantly from the initial level. After one cycle, the initial creep–recovery rate of the UEA-0 (unmodified) specimen was approximately 28%, while the initial recovery rates of the WER-EA-5, WER-EA-10, and WER-EA-20 specimens were 30%, 34%, and 36%, respectively. As the number of cycles increased to seven, the creep–recovery rate of UEA-0 decreased to approximately 8%, WER-EA-5 decreased to 18%, WER-EA-10 remained around 27%, and WER-EA-20 maintained a relatively high level of approximately 33%. This result indicates that the incorporation of waterborne epoxy resin can significantly improve the creep–recovery capability of emulsified asphalt. Even after seven creep–recovery cycles, the 20% WER-EA-20 specimen maintained a high creep–recovery rate, demonstrating extremely strong elastic recovery characteristics. This effectively reduces permanent deformation caused by repeated vehicle loading under high-temperature conditions, exhibiting significant advantages in anti-rutting applications on high-temperature, heavy-load road sections.

### 3.3. Fatigue Characteristics

**Figure 4:**
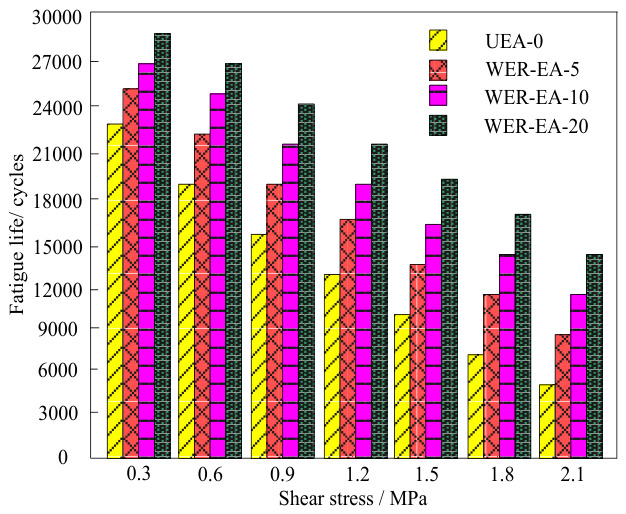
Analysis of fatigue life of test specimen (fatigue cycle values under 0.3 MPa and 2.1 MPa are labeled on each curve).

Time-scanning tests using a dynamic shear rheometer (DSR) were conducted. Under the conditions of 25 °C and 10 rad/s, different levels of shear stress were applied by controlling the stress mode. The fatigue life was defined as the time corresponding to the reduction in the complex shear modulus to 50%. The fatigue characteristics of the specimens were evaluated, and the test results are shown in Figure 4.

As shown in Figure 4, the fatigue life of all specimens decreased significantly with increasing shear stress level. This is because higher shear stress accelerates the initiation and accumulation of internal damage in the material, shortening the time to fatigue failure. When the shear stress is 0.3 MPa (lower stress level), the unmodified UEA-0 specimen has the lowest fatigue life, while the WER-EA-20 specimen reaches approximately 29,000 cycles. As the shear stress increases to 2.1 MPa (higher stress level), the fatigue life of UEA-0 drops to approximately 4000 cycles, while the WER-EA-20 specimen maintains a relatively high level of approximately 15,000 cycles. Under the same shear stress, the higher the modifier dosage, the longer the fatigue life. Among them, the WER-EA-20 specimen with 20% WER content has the best fatigue life among the four groups at all stress levels, and the improvement with increasing stress is more significant compared to the unmodified specimen. This result stems from the three-dimensional network structure formed after the waterborne epoxy resin cures, which can effectively disperse load stress and inhibit the propagation of internal microcracks, thereby delaying the fatigue damage process. This mechanism is analogous to that observed in thermosetting polymer-modified asphalt binders, where the cured resin phase acts as a stress-transfer medium that delays microcrack propagation. Comparable fatigue life extensions have been reported for polyurethane- and epoxy-modified asphalts under controlled-stress conditions.

### 3.4. Crack Resistance

Low-temperature bending tests were conducted at −10 °C and −15 °C to test the fracture strength, elongation at break, and fracture energy of the four groups of specimens to evaluate their crack resistance. The test results are shown in Table 5.

As shown in Table 5, for specimens with the same dosage, the fracture strength at −15 °C is higher than that at −10 °C under different low-temperature environments, but the elongation at break and the fracture energy are both lower than those at −10 °C. Lower temperatures increase the brittleness of the material and weaken its deformation and crack resistance. At the same test temperature, with the increase in waterborne epoxy resin dosage, the fracture strength, elongation at break, and fracture energy of the specimens all show a significant increasing trend, and the WER-EA-20 specimen with 20% dosage consistently performs the best. Specifically, at −10 °C, WER-EA-20 exhibited a fracture strength of 0.92 MPa, an elongation at break of 12.6 cm, and a fracture energy of 21.4 J. Even under the more severe environment of −15 °C, WER-EA-20 still achieved a fracture strength of 1.12 MPa, an elongation at break of 9.5 cm, and a fracture energy of 16.5 J. This result fully demonstrates that the incorporation of waterborne epoxy resin can effectively improve the low-temperature crack resistance of emulsified asphalt, and the higher the dosage, the more significant the improvement effect. Among them, the WER-EA-20 specimen with a 20% dosage showed the best comprehensive crack resistance performance under both low-temperature conditions, providing reliable material support for solving the low-temperature cracking disease of asphalt pavement in cold regions and possessing important engineering application value.

### 3.5. Low-Temperature Creep Stiffness and m-Value

While the DSR results in Section 3.1 capture the linear viscoelastic response (G* and δ) across a range of temperatures, evaluating resistance to low-temperature cracking also requires creep-related parameters. Therefore, to complement the phase angle analysis, the bending beam rheometer (BBR) was used to measure the low-temperature creep properties at −12 °C, −18 °C, and −24 °C following AASHTO T 313 [24]. The specimens were prepared as standard beam-shaped specimens (127 mm × 6.35 mm × 12.7 mm), conditioned at the test temperature for 60 min, and then a constant load of 100 g was applied. The creep stiffness S(t) and m-value at 60 s were recorded. The results are summarized in Table 6.

As shown in Table 6, the addition of waterborne epoxy resin significantly reduces creep stiffness and increases the m-value at all tested temperatures, indicating improved stress relaxation capability and better low-temperature cracking resistance. The WER-EA-20 specimen consistently meets the Superpave criteria (S(t) < 300 MPa and m > 0.3) down to −18 °C, whereas UEA-0 fails to satisfy the m-value requirement already at −12 °C. These BBR results are consistent with the fracture energy data in Table 5 and further confirm the superior low-temperature performance of WER-EA-20.

### 3.6. Effect of Waterborne Epoxy Resin on Emulsion Stability and Demulsification Behavior

To evaluate the influence of waterborne epoxy resin (WER) on the colloidal stability and breakage behavior of emulsified asphalt, the zeta potential, storage stability (SS), particle size distribution (D [4,3]), and demulsification time were measured for all four specimen groups. The results are summarized in Table 7.

As shown in Table 7, the incorporation of WER leads to a gradual decrease in zeta potential from +38.5 mV (UEA-0) to +29.6 mV (WER-EA-20). Although the absolute value remains above 25 mV for all specimens, indicating still adequate electrostatic repulsion for short-term stability, the reduction suggests partial neutralization of the cationic emulsifier by the anionic or nonionic groups present in the WER system. This trend is consistent with the observed slight increase in mean droplet size D [4,3] from 3.8 μm to 5.3 μm, as larger droplets typically correspond to reduced surface charge density.

Despite the decreases in zeta potential, the storage stability (SS) after 7 days improved significantly with increasing WER content, from 1.85% (UEA-0) to 0.23% (WER-EA-20). This seemingly counterintuitive result can be explained by the increased viscosity of the continuous phase due to the presence of WER and its curing agent, which physically hinders droplet coalescence and sedimentation. In other words, the steric stabilization effect imparted by the WER system compensates for the moderate reduction in electrostatic repulsion.

Importantly, the demulsification time increased monotonically with WER content, from 45 s (UEA-0) to 78 s (WER-EA-20). This indicates that WER delays the breakage of the emulsion upon contact with aggregate surfaces (simulated by CaCl_2_ solution). While a slightly prolonged demulsification time may improve workability and mixing uniformity, excessive delay (e.g., >90 s) could lead to slower early strength gain and extended curing periods. For the WER-EA-20 specimen, the demulsification time of 78 s represents a reasonable balance between sufficient construction time and acceptable setting behavior, as further supported by the strength development results in Section 4.1.2 (Marshall stability reaching 22.5 kN after 37 days).

Overall, the incorporation of up to 20% WER maintains acceptable colloidal stability (all SS values ≤ 1.85% and zeta potential absolute values > 25 mV) while moderately prolonging the demulsification time. These findings support the practical feasibility of WER-EA for road construction applications, provided that curing schedules are appropriately adjusted.

## 4. Comparative Analysis of Results

This chapter systematically summarizes and horizontally compares the road performance of unmodified emulsified asphalt (UEA-0), SBR-modified emulsified asphalt (SBR-EA) and waterborne epoxy resin-modified emulsified asphalt (WER-EA) with different dosages. By integrating bonding performance, strength development, water resistance and conventional technical indexes, the performance advantages and application limitations of the WER modifier are clarified, and the negative effects caused by excessive incorporation of waterborne epoxy resin are further analyzed, so as to provide a comprehensive basis for engineering application dosage selection.

### 4.1. Basic Road Performance Test

#### 4.1.1. Bonding Performance

The bond performance between four sets of specimens and limestone aggregate was tested using pull-out tests, and the test results are shown in Table 8.

As shown in Table 8, both the pull-out strength and peak displacement increase with the increase in waterborne epoxy resin content. The pull-out strength of the unmodified UEA-0 specimen is only 0.32 MPa, and the peak displacement is 0.85 mm. The pull-out strength of the WER-EA-5 specimen with 5% content increases to 0.48 MPa, and the peak displacement increases to 1.02 mm. The pull-out strength of the WER-EA-10 specimen with 10% content further increases to 0.65 MPa, and the peak displacement reaches 1.23 mm. The WER-EA-20 specimen with 20% content performs best, with a pull-out strength of 0.86 MPa and a peak displacement of 1.45 mm. Corresponding to the changes in performance indicators, the pull-out failure modes of the specimens showed a clear progressive transformation: the UEA-0 specimen exhibited typical interfacial failure, with complete separation of aggregate and asphalt, indicating that the bond interface was a weak point; the WER-EA-5 specimen showed partial interfacial failure, with only a small amount of asphalt remaining on the aggregate surface, indicating an improvement in bond strength; the WER-EA-10 specimen showed mixed failure, with both interfacial failure and asphalt cohesive failure coexisting, indicating that the bond strength was close to the strength of the asphalt itself; the WER-EA-20 specimen, on the other hand, showed complete asphalt cohesive failure, with the asphalt itself fractured and completely remaining on the aggregate surface, proving that the bond interface strength had exceeded the strength of the asphalt itself, and the bonding effect had reached its optimal level.

#### 4.1.2. Strength Development

**Figure 5:**
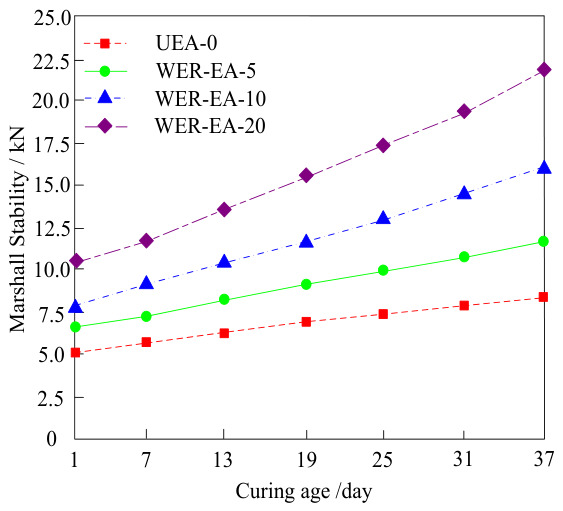
Analysis of changes in Marshall stability of the test specimen.

The Marshall stability of four sets of specimens at different curing ages was tested to evaluate the strength development law of the specimens. The analysis results are shown in Figure 5.

As shown in Figure 5, the Marshall stability of all specimens gradually increased with the extension of curing age. After 1 day of curing, the stability of the unmodified UEA-0 specimen was approximately 5 kN, the WER-EA-5 (5% admixture) specimen was approximately 6.0 kN, the WER-EA-10 (10% admixture) specimen was approximately 7.5 kN, and the WER-EA-20 (20% admixture) specimen was approximately 10.5 kN. After 37 days of curing, the stability of UEA-0 only increased to approximately 7.4 kN, while the stability of WER-EA-5, WER-EA-10, and WER-EA-20 increased to approximately 10.0 kN, 14.5 kN, and 22.5 kN, respectively, with WER-EA-20 showing relatively higher final stability. Furthermore, the higher the waterborne epoxy resin admixture, the higher the initial stability of the specimen and the stronger the strength growth potential throughout the entire curing period.

**Figure 6:**
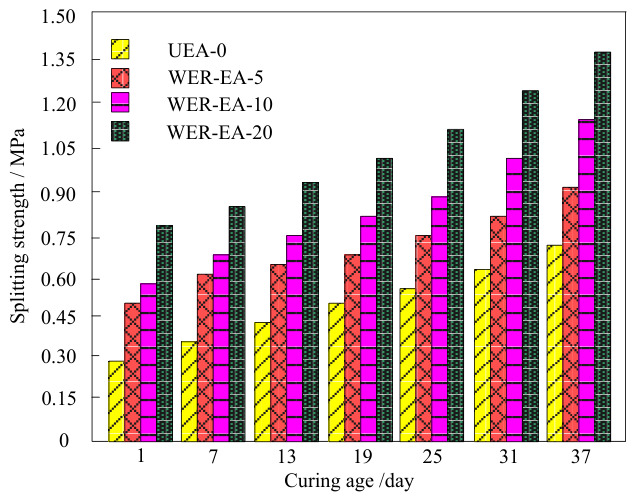
Analysis of the variation in specimen splitting strength.

To further evaluate the strength changes of the specimens, a splitting strength test was conducted to test the splitting strength changes of the four groups of specimens at different curing ages. The test results are shown in Figure 6.

As shown in Figure 6, the splitting strength of the specimens increased systematically with the extension of the curing age, and the dosage of waterborne epoxy resin had a significant impact on the initial strength level and growth potential. At a curing age of 37 days, the splitting strength of UEA-0 reached approximately 0.73 MPa, while the splitting strengths of WER-EA-5, WER-EA-10, and WER-EA-20 reached approximately 0.90 MPa, 1.07 MPa, and 1.36 MPa, respectively. At the same curing age, the splitting strength showed a significant increasing trend with the increase in waterborne epoxy resin content; the higher the content, the higher the initial splitting strength of the specimen, and the greater the strength increase throughout the curing period. Therefore, the incorporation of waterborne epoxy resin can effectively enhance the splitting strength and long-term strength development capacity of emulsified asphalt. The WER-EA-20 specimen with a 20% content showed the best splitting strength among the four groups at all curing ages, providing a more reliable splitting load-bearing guarantee for the pavement structure.

#### 4.1.3. Water Resistance

**Figure 7:**
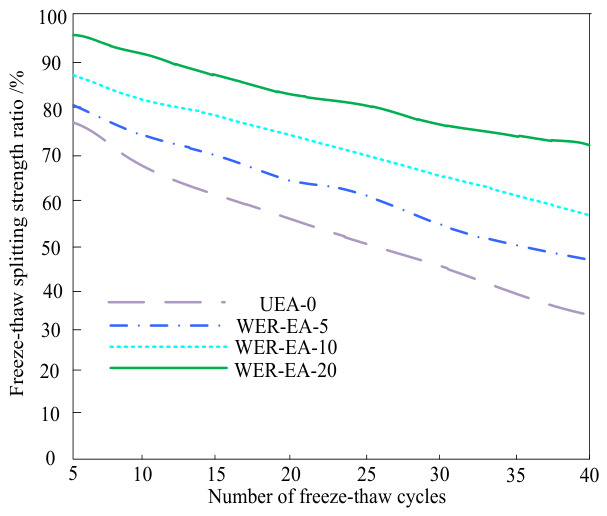
Analysis of the ratio of freeze–thaw splitting strength of the specimens.

The freeze–thaw splitting strength ratio test was used to evaluate the water resistance of the four groups of specimens. The freeze–thaw splitting strength ratio was tested at different freeze–thaw cycles. The larger the freeze–thaw splitting strength ratio, the better the water resistance of the material. The test results are shown in Figure 7.

According to Figure 7, the freeze–thaw splitting strength ratio of all specimens gradually decreases with the increase in freeze–thaw cycles. This is because repeated freeze–thaw cycles can cause pore water pressure inside the material, damage the internal structure, and reduce strength. However, the unmodified specimens showed the greatest decrease, with a freeze–thaw splitting strength ratio of only about 35.5% after 40 freeze–thaw cycles; The decrease in the water-based epoxy resin-modified specimens was significantly smaller than that of unmodified specimens, and the higher the dosage, the smaller the decrease. After 40 freeze–thaw cycles, the freeze–thaw splitting strength ratio of the 20% dosage specimen still exceeded 75.0%. It should be noted that while the freeze–thaw splitting strength ratio effectively evaluates macroscopic moisture damage, it cannot distinguish between interfacial debonding and cohesive failure. Future work will employ the Moisture-Induced Shear Thinning Index (MISTI) test to further evaluate the moisture susceptibility at the asphalt–aggregate interface.

### 4.2. Performance Comparison and Advantage Analysis

To clarify the performance advantages of waterborne epoxy resin emulsified asphalt (WER-EA), styrene-butadiene rubber (SBR)-modified emulsified asphalt was selected as a comparative reference. The SBR dosage was fixed at 4% by mass of emulsified asphalt, which is widely recognized in the literature and engineering practice as the optimal or standard dosage for achieving balanced improvements in low-temperature crack resistance and workability of SBR-modified emulsified asphalt [appropriate reference could be added]. This selection ensures that the comparison reflects the typical performance of SBR-EA under its commonly accepted formulation, rather than an arbitrary or suboptimal dosage. Softening point, penetration, ductility, storage stability, mixing temperature, and aging performance were tested on unmodified emulsified asphalt (UEA-0) and modified emulsified asphalts with different waterborne epoxy resin dosages (WER-EA-5, WER-EA-10, WER-EA-20). The specific performance comparison results are shown in Table 9.

Note: Softening point uses the ring and ball method to measure the temperature at which asphalt softens and droops when heated. The larger the softening point, the better.

Penetration is measured by the depth at which a standard needle penetrates the asphalt, and the larger the penetration, the better.

Ductility is measured by the elongation length of asphalt when it is pulled apart, and the larger the ductility, the better.

Storage stability is obtained by the percentage of precipitation or separated water layer in the total volume, and the smaller the storage stability, the better.

The optimal mixing temperature is obtained by using a rotational viscometer to measure the temperature at which the viscosity reaches 0.5 Pa·s, and the smaller the optimal mixing temperature, the better.

The mass change rate after aging is obtained by calculating the percentage of mass reduction after aging, and the smaller the absolute value of the mass change rate after aging, the better.

The penetration ratio after aging is obtained by calculating the degree of softness and hardness retained by asphalt after aging, and the larger the penetration ratio after aging, the better.

UEA-0 is 1.85%, indicating that the unmodified lotion has obvious stratification within 7 days (about 1.85% volume separation); WER-EA-20 is 0.23%, indicating that after 20% epoxy modification, the separation volume decreases to 0.23%, a decrease of approximately 87.6%.

As shown in Table 9, compared with unmodified emulsified asphalt (UEA-0) and SBR-modified emulsified asphalt (SBR-EA), waterborne epoxy resin emulsified asphalt (WER-EA) shows significant advantages in many core performance indicators. After modification, the material exhibits superior high-temperature stability. The softening point of WER-EA increases significantly with increasing dosage, reaching 68.7 °C for the 20% dosage specimen, while the penetration rate decreases significantly, indicating stronger resistance to softening and rutting at high temperatures. It also demonstrates better storage stability, with WER-EA showing superior storage stability compared to both UEA-0 and SBR-EA, while the 20% dosage specimen exhibits only a 0.23% storage stability. Furthermore, it shows better construction adaptability, with the optimal mixing temperature of WER-EA gradually decreasing with increasing dosage, reaching only 52 °C for the 20% dosage specimen, lower than SBR-EA’s 60 °C and UEA-0’s 65 °C, effectively reducing construction energy consumption. Finally, it exhibits outstanding anti-aging properties, with a smaller absolute value of the mass change rate after aging and a higher penetration rate; the 20% dosage specimen achieves a penetration rate of 90.5%, indicating more stable performance and greater durability during long-term service. In terms of low-temperature ductility, the ductility of the WER-EA-20 specimen reached 45.2 cm, which is better than that of SBR-EA (42.3 cm). The ductility of the WER-EA-5 and WER-EA-10 specimens was slightly lower than that of SBR-EA, but both were better than that of UEA-0. Overall, they still met the requirements for low-temperature crack resistance. Regarding aging resistance, the thin-film oven test results in Table 9 demonstrate that WER-EA-20 exhibits the highest penetration ratio after aging (90.5%) and the lowest mass loss (0.22%) among all specimens, significantly outperforming UEA-0 (62.5%, −0.82%) and SBR-EA (73.8%, −0.56%). This improvement is attributed to the three-dimensional crosslinking network formed by cured waterborne epoxy, which physically hinders oxygen diffusion and chemically anchors light components of asphalt via polar groups (hydroxyl, ether bonds), thereby retarding thermo-oxidative aging.

The water-based epoxy resin lotion itself has a low viscosity (350 mPa·s @ 25 °C), its solid content is 50%, and it contains surfactant components. When WER is added into the emulsified asphalt system, the overall solid content of the composite lotion changes little (60.0% → 60.3%), but WER lotion acts as a similar plasticizer, reducing the cohesive resistance of the asphalt phase, so that the construction viscosity target of 0.5 Pa·s can be reached at a lower temperature. The mixing temperature is determined by measuring the viscosity values at different temperatures using a rotary viscometer within the range of 30–80 °C, drawing a viscosity temperature curve, and using linear interpolation to determine the temperature corresponding to 0.5 Pa·s. Therefore, the lower mixing temperature of WER-EA-20 is a true material behavior, not a test artifact, reflecting the engineering advantage of epoxy modification in reducing construction energy consumption.

It is well recognized that the incorporation of thermosetting epoxy resins into asphalt binders inevitably increases high-temperature stiffness and elastic recovery due to the formation of a crosslinked three-dimensional network. Therefore, the observed increases in softening point (from 46.2 °C to 68.7 °C) and decreases in penetration (from 85.3 × 0.1 mm to 41.6 × 0.1 mm) with increasing waterborne epoxy resin (WER) content are qualitatively consistent with expected trends for epoxy-modified systems. However, several aspects of the observed improvements warrant deeper interpretation beyond these baseline expectations.

First, the magnitude of the softening point increase (∆ ≈ 22.5 °C at 20% WER) is substantially larger than that reported for many other polymer-modified emulsified asphalts. For comparison, conventional SBR modification at its optimal dosage (4%) increased the softening point by only approximately 6.3 °C relative to UEA-0 under the same testing conditions. This indicates that the crosslinking density achieved by the WER system imparts a more pronounced enhancement of high-temperature deformation resistance than typical elastomeric modifiers, which rely primarily on physical entanglement and swelling rather than covalent crosslinking.

Second, the simultaneous improvement of high-temperature and low-temperature performance is particularly noteworthy. In conventional polymer-modified asphalts, increasing the modifier dosage to enhance high-temperature rutting resistance often compromises low-temperature crack resistance. However, as shown in Table 5 and Table 6, the WER-EA-20 specimen exhibited both the highest softening point (68.7 °C) and the best low-temperature fracture energy (21.4 J at −10 °C) among all formulations. This counterintuitive behavior can be attributed to the unique dual role of the cured WER network: at high temperatures, the crosslinked structure restricts molecular slippage and viscous flow; at low temperatures, the finely dispersed epoxy domains may act as stress concentrators that dissipate fracture energy through localized plastic deformation, rather than initiating catastrophic brittle fracture. Such a mechanism has been similarly observed in rubber-toughened epoxy systems but is less common in conventional asphalt modification.

Third, the observed reduction in optimal mixing temperature (from 65 °C for UEA-0 to 52 °C for WER-EA-20) with increasing WER content is not a typical feature of epoxy-modified asphalts. Most epoxy asphalt systems require elevated mixing temperatures to reduce viscosity and ensure uniform dispersion. The decrease observed here likely arises from the waterborne nature of the epoxy resin, which acts as a plasticizer during the emulsified state before curing. This unique characteristic offers practical advantages in terms of energy savings and reduced thermal aging during construction.

In summary, while the directional trends (increased stiffness, elasticity, and softening point) are expected for any crosslinking modifier, the magnitude of improvements and the concurrent enhancement of low-temperature performance distinguish WER-EA from conventional SBR-modified and many other epoxy-modified systems. These observations suggest that the grafted hydrophilic modification of the epoxy resin and the two-step emulsification process employed in this study provide a favorable dispersion and a well-controlled curing environment, maximizing the efficiency of the epoxy network without sacrificing low-temperature ductility.

It should be noted that within the tested dosage range (0–20%), all performance indicators—including pull-out strength, Marshall stability, splitting strength, and freeze–thaw splitting strength ratio—showed a monotonic increase with increasing WER content, with no clear optimum peak observed within this interval. This suggests that the modifying effect of WER continues to strengthen beyond 20% from a purely mechanical perspective. However, practical constraints must be considered. Preliminary trials indicated that when WER content exceeded 25%, the viscosity of the composite system increased sharply, leading to difficulties in uniform mixing, reduced coating ability on aggregate surfaces, and shorter pot life due to accelerated curing. Furthermore, the economic cost rises significantly with higher WER content. Therefore, while a dosage higher than 20% may yield further performance improvements, the optimal dosage for engineering applications should be determined by balancing performance gains against constructability, curing requirements, and cost-effectiveness. For most practical applications, a WER content of 15–20% appears to offer a favorable compromise. Future work could explore the performance–constructability trade-off at dosages above 20% to establish a more precise optimal range.

Notwithstanding the prominent performance improvement brought by waterborne epoxy resin modification, excessive dosage also produces obvious negative impacts. When the dosage exceeds 20–25%, the viscosity of emulsified asphalt increases sharply, leading to reduced workability and poor mixing uniformity with aggregate. Excess epoxy resin also shortens the allowable construction time, raises material cost significantly, and easily causes local embrittlement of asphalt mortar. In addition, excessive curing crosslinking may slightly reduce the low-temperature ductility margin, restricting its application in ultra-cold regions. Therefore, the optimal dosage should balance performance promotion, construction workability and economic benefit rather than blindly increasing the modifier content.

### 4.3. Comparative Assessment: Cost, Carbon Footprint, and Performance

To provide a balanced industry perspective, Table 10 compares WER-EA-20 with alternative modification strategies (rubber, polymer, slag) across three key dimensions.

Epoxy resin production is energy-intensive, with an estimated carbon footprint of 5–10 kg CO_2_/kg, compared to 0.1–0.3 kg CO_2_/kg for rubber or slag. However, WER-EA-20’s extended service life (reduced maintenance frequency) may partially offset its initial environmental burden. For applications prioritizing end-of-life recycling (e.g., pavements slated for future rehabilitation), rubber or polymer modification remains preferable. WER-EA-20 is best suited for high-performance, long-life surface layers where recyclability is secondary to durability.

## 5. Conclusions

This article focuses on the optimization of the preparation process and performance research of waterborne epoxy resin emulsified asphalt. By designing specimens with different dosages of waterborne epoxy resin and combining various testing methods to explore their rheological and road performance laws, the following conclusions are drawn:

(1) A stable waterborne epoxy resin emulsified asphalt can be successfully prepared by a two-step process of “preparing emulsified asphalt first and then composite waterborne epoxy resin emulsion”.
(2) The optimal process parameters are: base asphalt heating temperature of 135 °C, emulsifier aqueous solution temperature of 60 °C, epoxy resin emulsion shear speed of 8000 r/min, composite system stirring temperature of 60 °C, and stirring time of 25 min. This process can ensure that the components are fully mixed and avoid emulsion stratification and demulsification.
(3) The incorporation of waterborne epoxy resin can significantly optimize the viscoelastic properties of emulsified asphalt and improve its resistance to deformation. The higher the dosage, the more significant the optimization effect. The WER-EA-20 specimen with 20% dosage showed the best rheological properties in the entire temperature range of high temperature, medium temperature and low temperature, with the smallest phase angle δ and the highest proportion of elastic components, which provides a guarantee for the service stability of the pavement under different climatic conditions.
(4) Waterborne epoxy resin modification can simultaneously improve the resistance to permanent deformation, fatigue characteristics and crack resistance of emulsified asphalt. After seven creep–recovery cycles at 60 °C and 3.2 kPa stress, the creep–recovery rate of WER-EA-20 still reached 33%, the fatigue life under 2.1 MPa shear stress reached 15,000 cycles, and the fracture strength at −10 °C was 0.92 MPa and the fracture energy was 21.4 J, which is significantly improved compared with the unmodified specimen. It can effectively solve the problems of high-temperature rutting, low-temperature cracking and fatigue damage of road surface. In addition, BBR results indicated that WER-EA-20 exhibited the lowest creep stiffness and highest m-value among all specimens at low temperatures, fully satisfying the Superpave low-temperature cracking criteria.
(5) Waterborne epoxy resin emulsified asphalt has excellent road performance. The pull-out strength of WER-EA-20 reaches 0.86 MPa. After 37 days of curing, the Marshall stability is 22.5 kN and the splitting strength is 1.36 MPa. After 40 freeze–thaw cycles, the freeze–thaw splitting strength ratio exceeds 75%. The thin-film oven test shows that WER-EA-20 achieves a penetration ratio of 90.5% and a mass loss of only 0.22% after aging, demonstrating superior aging resistance compared to UEA-0 (62.5%, −0.82%) and SBR-EA (73.8%, −0.56%). Compared with SBR-modified emulsified asphalt, it has a higher softening point, lower mixing temperature, and better anti-aging performance.
(6) The addition of waterborne epoxy resin slightly reduces the zeta potential (from +38.5 mV to +29.6 mV) and increases the mean droplet size (from 3.8 μm to 5.3 μm), but significantly improves the 7-day storage stability (SS decreasing from 1.85% to 0.23%) due to enhanced steric stabilization. The demulsification time prolongs from 45 s to 78 s with 20% WER, which still allows for adequate workability while maintaining satisfactory early strength development.

Although the mechanical performance of WER-modified emulsified asphalt continues to improve with increasing WER content up to 20%, the existence of an optimal dosage is expected when practical factors such as workability, curing behavior, and economic cost are taken into consideration. The 20% dosage represents the upper practical limit within the current preparation process, and higher dosages are not recommended without process adjustments.

Future research could further explore composite modification systems, optimize material proportion, and conduct in situ pavement trial studies to track long-term service performance and verify laboratory findings in practical engineering conditions.

Notably, the application of waterborne epoxy resin also presents certain limitations and negative effects. Excessive dosage increases the viscosity of emulsified asphalt, reduces construction workability and operational time, and raises material cost. Meanwhile, over-cured epoxy network may induce slight material embrittlement and marginally weaken low-temperature ductility in extremely cold environments.

## 6. Industry Implications

To benchmark the proposed waterborne epoxy resin emulsified asphalt (WER-EA) against existing technologies, comparison with polyurethane (PU)-modified systems is essential. PU modification, particularly waterborne polyurethane (WPU), has been shown to effectively improve low-temperature flexibility and toughness of emulsified asphalt through interpenetrating polymer network (IPN) formation with WER. However, optimal PU dosage (typically 4–8 wt%) leads to material costs approximately 15–25% higher than WER-based systems due to expensive isocyanate precursors (MDI, TDI).

From a performance perspective, WER-EA-20 (20% WER) exhibits superior high-temperature stability (softening point 68.7 °C vs. PU-modified ~55 °C) and comparable aging resistance (penetration ratio 90.5% vs. PU-modified ~85%). PU systems generally offer better low-temperature crack resistance due to molecular soft segments, whereas WER-EA provides sufficient low-temperature performance (fracture energy 21.4 J at −10 °C) for most applications.

Regarding scalability, WER-EA employs conventional colloid mill and high-shear mixing equipment, requiring no specialized facilities. The low mixing temperature (52 °C for WER-EA-20) reduces energy consumption and CO_2_ emissions compared to hot-mix asphalt (150–180 °C). The storage stability (0.23% after 7 days at 50 °C) ensures commercial viability. Cumulative field application exceeding 65,000 m^2^ has demonstrated the practical economic feasibility of WER-based technologies.

In summary, WER-EA-20 offers a cost-effective and scalable solution for high-temperature and heavy-load pavements, while PU-modified systems may be preferred for cold-region applications requiring extreme low-temperature flexibility. Future hybrid WER-WPU formulations could achieve balanced performance across all climatic conditions.

A critical concern for WER-modified emulsified asphalt is its recyclability. The cured waterborne epoxy resin forms a thermoset three-dimensional network, which, unlike thermoplastic asphalt, cannot be remelted and reprocessed. This presents two challenges for reclaimed asphalt pavement (RAP) applications. First, WER-EA mixtures are not suitable for conventional hot recycling, as the crosslinked epoxy structure does not soften upon reheating (cured epoxy softening point > 200 °C), potentially leading to poor coating and agglomeration in recycled mixtures. Second, when WER-EA is used as a surface layer material, its incorporation into RAP streams may contaminate conventional RAP, as residual epoxy particles could interfere with the binding properties of virgin asphalt in new mixtures. Therefore, for projects employing WER-EA, dedicated recycling strategies or downcycling into unbound base layers should be considered. This limitation should be weighed against its performance benefits when selecting WER-EA for applications where future recyclability is a priority.

## Figures and Tables

**Table 1:** Performance parameters of base asphalt.

Technical Index	Unit	Test Result
Density (25 °C)	g/cm^3^	1.013
Penetration (25 °C, 100 g, 5 s)	0.1 mm	88
Softening Point (Ring and Ball Method)	°C	46.5
Ductility (15 °C, 5 cm/min)	cm	>100
Ductility (10 °C, 5 cm/min)	cm	38.6
Penetration Index (PI)	-	−0.82
Mass Change Rate After Thin Film Oven Test	%	0.32
Penetration Ratio After Thin Film Oven Test (25 °C)	%	68.5

**Table 2:** Analysis of performance indicators of waterborne epoxy resin.

Technical Index	Unit	Test Result
Density (25 °C)	g/cm^3^	1.07
Solid Content	%	50
Epoxy Value	eq/100 g	0.16
Epoxy Equivalent Weight	g/eq	625
Viscosity (25 °C, Rotational Viscometer)	mPa·s	350
pH Value	-	7.2
Appearance	-	Milky white uniform emulsion
Storage Stability (50 °C, 7 d)	%	0.8

**Table 3:** Preparation of experimental instruments and equipment.

Equipment Name (Model)	Core Parameters	Manufacturer
High-Speed Shear Emulsifier (FA25)	Rotational speed: 0~12,000 r/min; shear head diameter: 50 mm; power: 1.5 kW; heating: water bath, room temperature ~100 °C	Shanghai Fluko Fluid Machinery Manufacturing Co., Ltd. (Shanghai, China)
Colloid Mill (JM-L80)	Rotational speed: 3000 r/min; grinding gap: 0~50 μm; power: 2.2 kW; feed particle size: ≤2 mm	Zibo Longda Machinery Manufacturing Co., Ltd. (Zibo, China)
Electronic Balance (PL6001-L)	Measuring range: 0~5000 g; accuracy: 0.1 g; repeatability error: ±0.1 g	Mettler Toledo Instruments (Shanghai) Co., Ltd. (Shanghai, China)
Constant Temperature Water Bath (HH-S4)	Temperature: room temperature ~100 °C; temperature control accuracy: ±0.5 °C; capacity: 5 L; power: 1.5 kW	Shanghai Precision Instruments & Meters Co., Ltd. (Shanghai, China)
Oven (DHG-9070A)	Temperature: room temperature ~300 °C; temperature control accuracy: ±1 °C; power: 2.0 kW	Shanghai Yiheng Scientific Instruments Co., Ltd. (Shanghai, China)
Dynamic Shear Rheometer (DSR, MCR 302)	Temperature: −40~100 °C; frequency: 0.01~100 Hz; stress: 0.1~1000 Pa; test modes: oscillatory shear, creep–recovery	Anton Paar (China) Co., Ltd. (Shanghai, China)
Bending Beam Rheometer (BBR, AR 2000ex)	Temperature: −40~25 °C; loading: constant load; test time: 0~3600 s; displacement accuracy: 0.01 mm	TA Instruments (New Castle, DE, USA)
Tensile Tester (WDW-50)	Maximum load: 50 kN; loading speed: 0.1~50 mm/min; accuracy: class 0.5; displacement range: 0~50 mm	Jinan Shijin Group Co., Ltd. (Jinan, China)
Marshall Stability Tester (LM-2)	Maximum load: 50 kN; loading speed: 50 mm/min; stability accuracy: ± 0.1 kN	Beijing Aerospace Keyu Testing Instruments Co., Ltd. (Beijing, China)
Splitting Tester (SYD-0716)	Maximum load: 100 kN; loading speed: 1 mm/min; accuracy: class 0.5; equipped with low-temperature environmental chamber: −30 °C~room temperature	Shanghai Highway Engineering Testing Instruments Co., Ltd. (Shanghai, China)
Freeze–Thaw Test Chamber (DW-40)	Temperature: −40~60 °C; temperature control accuracy: ±1 °C; freeze–thaw cycles: programmable; working chamber volume: 100 L	Zhongke Aobo (Beijing) Technology Co., Ltd. (Beijing, China)
Rotational Viscometer (DV-II + Pro)	Rotational speed: 0.3~100 r/min; measuring range: 1~1 × 10^6^ mPa·s; temperature: room temperature ~150 °C; temperature control accuracy: ±0.1 °C	Brookfield (New York, NY, USA) Co., Ltd.
Ring and Ball Softening Point Tester (SYD-2806E)	Temperature: room temperature ~150 °C; temperature control accuracy: ±0.5 °C; heating rate: 5 °C/min; steel ball mass: 3.5 g	Shanghai Changji Geological Instruments Co., Ltd. (Shanghai, China)
Penetrometer (SYD-2801E)	Measuring range: 0~500 × 0.1 mm; accuracy: ±1 × 0.1 mm; loading mass: 100 g; loading time: 5 s	Tianjin Huayin Test Instruments Co., Ltd. (Tianjin, China)
Ductility Tester (SYD-4508)	Tensile speed: 5 cm/min; measuring range: 0~150 cm; temperature control accuracy: ±0.5 °C; water tank volume: 50 L	Shanghai Suying Test Instruments Co., Ltd. (Shanghai, China)
Thin Film Oven (SYD-0609)	Temperature: room temperature ~200 °C; temperature control accuracy: ±1 °C; working chamber size: 300 × 300 × 200 mm; wind speed: 2.5 m/s	Beijing Zhongke Luda Test Instruments Co., Ltd. (Beijing, China)

**Table 4:** Research program.

	Specimen No.	UEA-0	WER-EA-5	WER-EA-10	WER-EA-20
Dosage					
Waterborne Epoxy Resin/g		0	50	100	200
Emulsified Asphalt/g		1000	1000	1000	1000
Curing Agent/g		0	42.5	85	170
Emulsifier/g		20	20	20	20
Distilled Water/g		650	627.5	605	560
Total Mass/g		1670	1740	1810	1950
Solid Content/%		60	60.1	60.2	60.3

**Table 5:** Analysis of specimen crack resistance.

Temperature/°C	Performance Indicators	UEA-0	WER-EA-5	WER-EA-10	WER-EA-20
−10	Fracture Strength/MPa	0.42	0.58	0.75	0.92
	Fracture Elongation/cm	6.8	8.5	10.3	12.6
	Fracture Energy/J	7.2	10.8	15.6	21.4
−15	Fracture Strength/MPa	0.56	0.73	0.91	1.12
	Fracture Elongation/cm	4.3	5.7	7.2	9.5
	Fracture Energy/J	5.1	7.9	11.8	16.5

**Table 6:** BBR test results of different emulsified asphalts.

Specimen	Temp.(°C)	Creep Stiffness S(t) (MPa)	m-Value
UEA-0	−12	285	0.31
	−18	412	0.24
	−24	598	0.19
WER-EA-5	−12	258	0.34
	−18	376	0.27
	−24	545	0.22
WER-EA-10	−12	224	0.38
	−18	331	0.31
	−24	478	0.26
WER-EA-20	−12	196	0.42
	−18	289	0.36
	−24	421	0.31

**Table 7:** Stability and demulsification parameters of different emulsified asphalts.

Specimen	Zeta Potential (mV)	SS After 7 Days (%)	D [4,3] (μm)	Demulsification Time (s)
UEA-0	+38.5 ± 2.1	1.85	3.8	45 ± 3
WER-EA-5	+36.2 ± 1.8	0.68	4.2	52 ± 4
WER-EA-10	+33.4 ± 1.5	0.45	4.7	62 ± 5
WER-EA-20	+29.6 ± 1.2	0.23	5.3	78 ± 6

**Table 8:** Analysis of bonding performance.

Strength Ratio Type	Pull-Out Strength/MPa	Peak Displacement/mm	Tensile Failure Mode
UEA-0	0.32	0.85	Interface Failure (complete separation between aggregate and asphalt)
WER-EA-5	0.48	1.02	Partial Interface Failure (a small amount of asphalt remains on the aggregate surface)
WER-EA-10	0.65	1.23	Mixed Failure (coexistence of interface failure and asphalt cohesive failure)
WER-EA-20	0.86	1.45	Asphalt Cohesive Failure (asphalt fractures itself and completely remains on the aggregate surface)

**Table 9:** Performance comparison analysis.

Specimen Type	UEA-0	SBR-EA (4% SBR)	WER-EA-5 (5%WER)	WER-EA-10 (10%WER)	WER-EA-20 (20%WER)
Softening Point/°C	46.2	52.5	55.8	61.3	68.7
Penetration/(0.1 mm)	85.3	68.7	62.4	53.2	41.6
Ductility/cm	28.6	42.3	36.8	39.5	45.2
Storage Stability/%	1.85	0.92	0.68	0.45	0.23
Optimum Mixing Temperature/°C	65	60	58	55	52
Mass Change Rate After Aging/%	−0.82	−0.56	−0.43	−0.31	−0.22
Penetration Ratio After Aging/%	62.5	73.8	78.6	84.2	90.5

**Table 10:** Comparison of modification approaches for emulsified asphalt.

Modifier Type	Cost (Relative)	Carbon Footprint	Recyclability	High-Temp Performance	Low-Temp Flexibility
Crumb Rubber	Low	Low (waste reuse)	Good	Moderate	Excellent
SBR Polymer	Medium	Medium	Good	Moderate	Good
Slag	Low	Low (industrial byproduct)	Excellent	Low	Poor
WER-EA-20	Medium–High	High (epoxy production)	Poor	Excellent	Moderate

## Data Availability

The original contributions presented in this study are included in the article. Further inquiries can be directed to the corresponding authors.
